# Rapidly measuring spatial accessibility of COVID-19 healthcare resources: a case study of Illinois, USA

**DOI:** 10.1186/s12942-020-00229-x

**Published:** 2020-09-14

**Authors:** Jeon-Young Kang, Alexander Michels, Fangzheng Lyu, Shaohua Wang, Nelson Agbodo, Vincent L. Freeman, Shaowen Wang

**Affiliations:** 1 CyberGIS Center for Advanced Digital and Spatial Studies, University of Illinois at Urbana-Champaign, Urbana, IL USA; 2 Department of Geography and Geographic Information Science, University of Illinois at Urbana-Champaign, Urbana, IL USA; 3 Illinois Informatics Institute, University of Illinois at Urbana-Champaign, Urbana, IL USA; 4 Division of Health Data and Policy, Illinois Department of Public Health, Springfield, IL USA; 5 Division of Epidemiology and Biostatistics, School of Public Health, University of Illinois at Chicago, Chicago, IL USA

**Keywords:** COVID-19, CyberGIS, Social vulnerability, Spatial accessibility, Spatial analysis

## Abstract

**Background:**

The severe acute respiratory syndrome coronavirus 2 (SARS-CoV-2), causing the coronavirus disease 2019 (COVID-19) pandemic, has infected millions of people and caused hundreds of thousands of deaths. While COVID-19 has overwhelmed healthcare resources (e.g., healthcare personnel, testing resources, hospital beds, and ventilators) in a number of countries, limited research has been conducted to understand spatial accessibility of such resources. This study fills this gap by rapidly measuring the spatial accessibility of COVID-19 healthcare resources with a particular focus on Illinois, USA.

**Method:**

The rapid measurement is achieved by resolving computational intensity of an enhanced two-step floating catchment area (E2SFCA) method through a parallel computing strategy based on cyberGIS (cyber geographic information science and systems). The E2SFCA has two major steps. First, it calculates a bed-to-population ratio for each hospital location. Second, it sums these ratios for residential locations where hospital locations overlap.

**Results:**

The comparison of the spatial accessibility measures for COVID-19 patients to those of population at risk identifies which geographic areas need additional healthcare resources to improve access. The results also help delineate the areas that may face a COVID-19-induced shortage of healthcare resources. The Chicagoland, particularly the southern Chicago, shows an additional need for resources. This study also identified vulnerable population residing in the areas with low spatial accessibility in Chicago.

**Conclusion:**

Rapidly measuring spatial accessibility of healthcare resources provides an improved understanding of how well the healthcare infrastructure is equipped to save people’s lives during the COVID-19 pandemic. The findings are relevant for policymakers and public health practitioners to allocate existing healthcare resources or distribute new resources for maximum access to health services.

## Introduction

A novel coronavirus disease (COVID-19), caused by severe acute respiratory syndrome coronavirus 2 (SARS-CoV-2), has widely spread worldwide. As of April 10, 2020, about 1.6 million COVID-19 cases have been confirmed in the world; and in the United States alone, over 475,000 people have been infected with more than 17,000 deaths. Among the infected cases, hundreds of thousands of people are hospitalized. The COVID-19 pandemic has exceeded the capacities of healthcare resources, including for example healthcare personnel, testing resources, hospital beds, beds in intensive care units (ICUs) and ventilators [1–3]. This increased demand for healthcare resources has caused or exacerbated health disparities in access to healthcare [4]. To resolve this demand and mitigate the coronavirus, the state of Illinois in the USA issued a Disaster Proclamation on March 9, 2020 with the following subsequent actions: restricted nursing home visit started on March 11; bars and restaurant close for on-site consumption started on March 16; remote learning for students started on March 17; and a Stay-at-Home order started on March 21.

A key requirement for our research is to dynamically and rapidly measure spatial accessibility of COVID-19 healthcare resources. Our approach fulfills this requirement by achieving the integration of innovative computational strategies and cutting-edge cyberGIS (cyber geographic information science and systems) (Wang [28]) capabilities to conduct computationally intensive spatial accessibility analysis in a timely manner and make analytical outcome dynamically available for decision-making support. While there exist many geospatial data dashboards and portals (e.g., COVID-19 Dashboard by Johns Hopkins University [5], an interactive map for COVID-19 hospital capacity by the Harvard Global Health Institute [6], and CoronaVis by the Data Analysis and Visualization Group at the University of Konstanz in Germany [7] providing useful information such as the number of cases, deaths, tests, and current status of healthcare resources [8]), none of the existing approaches has achieved the integration aimed in this research.

Oftentimes, SARS-CoV-2 infection cases and related healthcare resources in demand are not spatially equitably distributed [9]. In other words, spatial mismatches need to be resolved between healthcare resource availability and the needs of COVID-19 patients and population at risk. Identifying these mismatches is crucial for allocating healthcare resources efficiently and effectively [10]. Ensuring healthcare services are equitably accessible to communities has been a major focus in the global context of healthcare policy making [11]. Understanding spatial accessibility of healthcare services is important to the development and allocation of local healthcare services [12]. For example, identifying health professional shortage areas and medically underserved populations has been done extensively by the US Department of Health and Human Services (HHS) to determine eligibility for federal healthcare resources [13]. As an example at the state level, the Illinois Health Facilities and Services Review Board (HFSRB) has highlighted the importance of a comprehensive health care delivery system that ensures spatial proximity of healthcare facilities and accessibility of their services and equipment to communities based on assessment of needs [14]. The Illinois Department of Public Health (IDPH) has committed significant efforts on improving hospitals’ bed capacity and COVID-19 related resources [15]. Therefore, it is important to understand how to conduct rigorous spatial accessibility analysis to support healthcare decision making across multiple spatial scales.

Measuring spatial accessibility to healthcare resources has long been of interest to public health research. Examples include healthcare access for seniors [16, 17], disabled people [18], cancer-specific survivals [19, 20], examining spatial accessibility among populations with multiple transportation modes [21, 22], and access to specific health care treatments such as mammograms [23]. Spatial accessibility of healthcare resources can be measured by spatial interactions between the amount of supplies (e.g., the number of hospital beds or physicians) and demands along with the distance and travel time between the locations of healthcare resources and those of residential areas.

A commonly used method for measuring spatial accessibility is the two-step floating catchment area (2SFCA) method [24]. Particularly, the enhanced two-step floating catchment area (E2SFCA) method accounts for distance decay [25]. E2SFCA uses travel time for a given mode of transportation to calculate the areas that are within 10, 20, and 30 min of supply locations. For large study areas such as the state of Illinois in the USA, calculating catchment areas on a road network has high computational intensity [26]. CyberGIS—defined as geographic information science and systems based on advanced cyberinfrastructure—is well suited to resolve this type of computational intensity through high-performance parallel computing [27–29].

Motivated to address this computationally intensity challenge, this study aims to rapidly measure the spatial accessibility of healthcare resources in Illinois. Specifically, we seek to answer the following three research questions: (1) to what extent Illinois residents have access to healthcare resources during the COVID-19 pandemic? (2) which geographic areas have abundant resources and which areas have insufficient resources? and (3) to what extent the spatial accessibility is associated with socioeconomic and demographic characteristics? To answer these questions, spatial accessibility was measured based on travel time between the locations of residence and healthcare resources in the context of COVID-19 patients and population at risk (i.e., people aged over 50 years). As of August 14, 2020, there are 7721 COVID-19 deaths in Illinois, of which 95% (7296) are residents aged more than 50 years (IDPH [30]).

We have developed a parallel enhanced two-step floating catchment area (P-E2SFCA) method based on CyberGIS-Jupyter—a cyberGIS framework for achieving data-intensive, reproducible, and scalable geospatial analytics using Jupyter Notebook [31–34]. In our study, the demand of healthcare resources comes from either COVID-19 patients or vulnerable population. We compared spatial accessibility of healthcare between COVID-19 patients and population at risk. Social vulnerability is often assessed to understand population’s susceptibility to external stresses, such as natural disasters [35, 36] and disease outbreaks [37, 38] including COVID-19 [39, 40]. We examine the socioeconomic and demographic characteristics in the areas with high and low accessibility, by leveraging the social vulnerability index (SVI) developed by the U.S. Centers for Disease Control and Prevention (CDC) [41]. Our analysis provides insights for decision making to optimize the allocation of COVID-19-related healthcare resources, such as hospital beds, testing resources, healthcare personnel, ICU beds and ventilators. The rest of the paper is organized as follows. The “Data and method” section describes the data and method used in the study. Section 3 summarizes and evaluates the results obtained from the P-E2SFCA method. Section 4 concludes with a discussion about policy implications and future directions.

## Data and method

### Study area and data

This study focuses on spatial accessibility of healthcare resources for the general population and COVID-19 patients in Illinois, USA. We used four types of datasets, summarized as follows: (1) hospital dataset, including the number of beds in intensive care units (ICUs) and the number of ventilators in each hospital, (2) COVID-19 confirmed case dataset, (3) residential dataset, and (4) road network dataset. The hospital dataset was provided by IDPH. The U.S. Homeland Infrastructure Foundation Level Data (HIFLD) Subcommittee provides national-level geospatial data that can be used in supporting community preparedness and research. The COVID-19 confirmed case dataset at the zip code level is provided by IDPH [30]. The residential dataset was obtained from the United States Census Bureau. We used an Application Programming Interface (API) call for pulling data from the 2018 American Community Survey 5-year detail table for each census tract in the state of Illinois, USA. The road network dataset was retrieved using a Python package OSMnx [42], which helped us to download and analyze street networks from the OpenStreetMap.

**Fig. 1:**
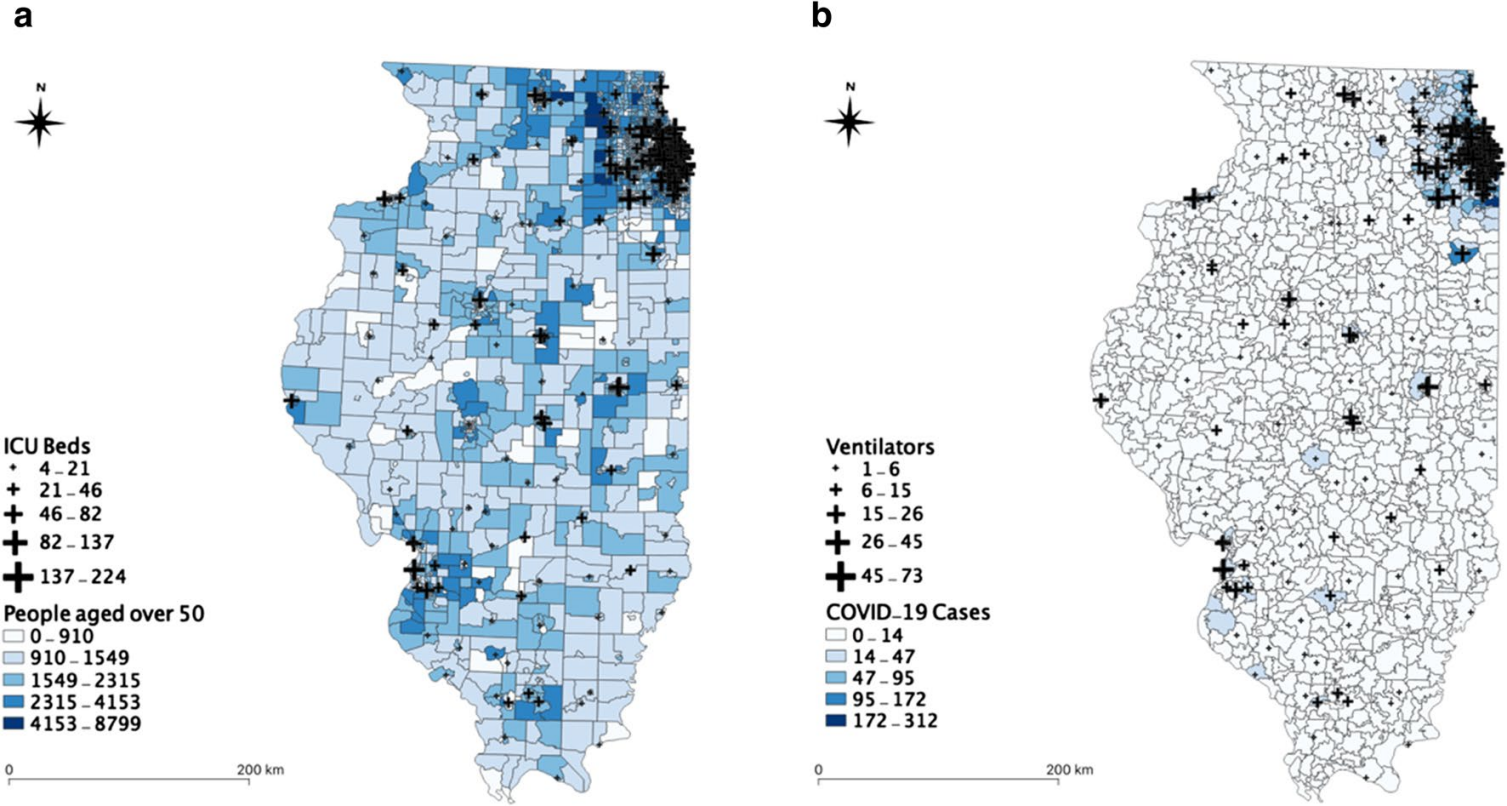
Datasets of hospital, population, COVID-19 cases in Illinois: **a** spatial distributions of ICU beds and population at risk; **b** spatial distributions of ventilators and confirmed COVID-19 cases

**Fig. 2:**
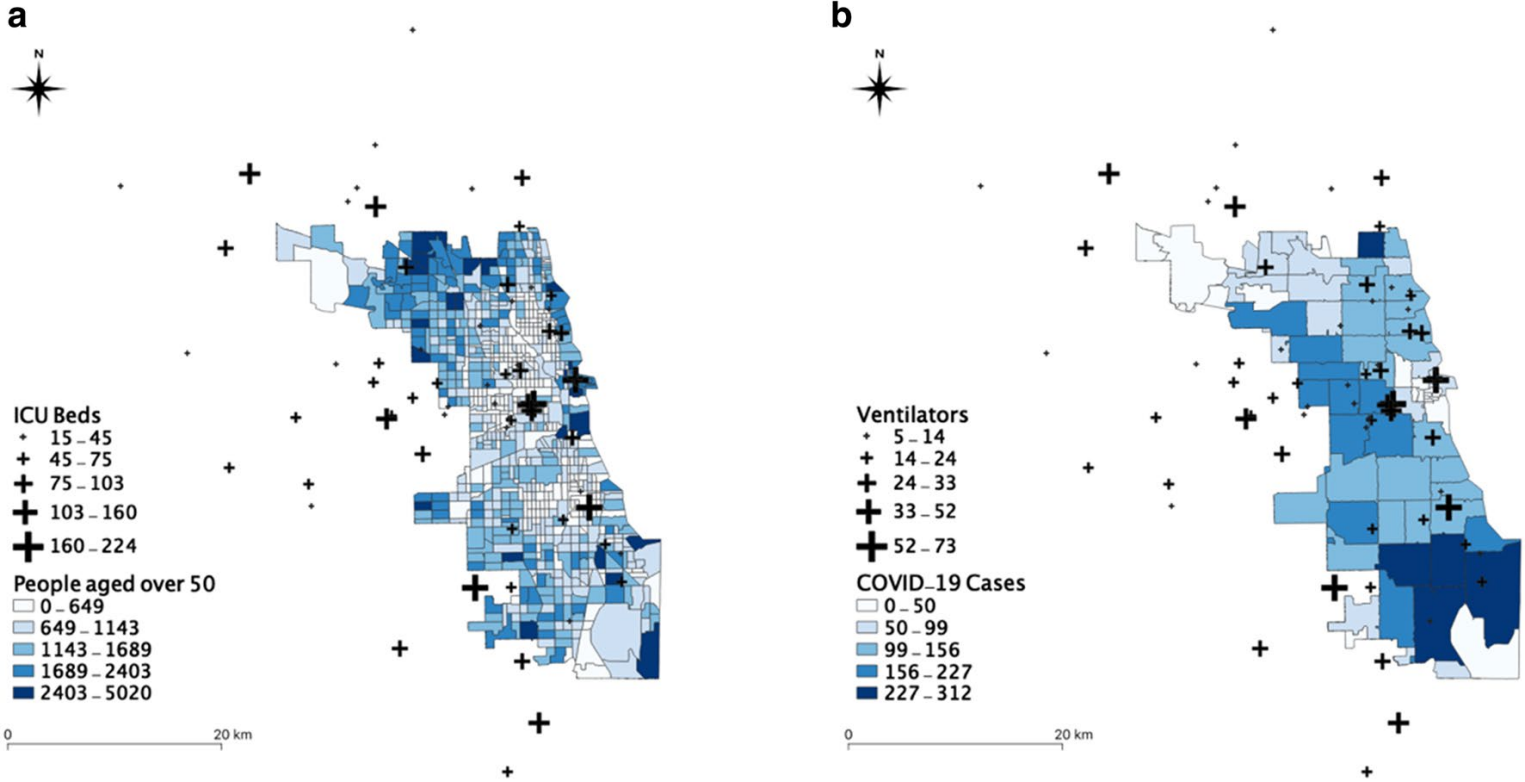
Datasets of hospitals, population, COVID-19 cases in Chicago, Illinois: **a** spatial distributions of ICU beds and population at risk, and **b** spatial distributions of ventilators and confirmed COVID-19 cases. To avoid the edge effects, we included hospitals located within 15 miles from the Chicago boundary. 15 miles are considered based on estimated average travel time of about 30 min [24]

In Illinois, there are 183 hospitals from which the information of beds is available in the dataset. We excluded some hospitals, including military, children, psychiatric, and rehabilitation in this study, because these types of hospitals may not provide health services to COVID-19 patients. Figure 1a illustrates the spatial distribution of ICU beds with the population across Illinois. Figure 1b shows the spatial distribution of ventilators with the COVID-19 confirmed cases in Illinois as of April 10, 2020. Obviously, the population in Illinois is dense in the Chicago area, and significant clustering of the COVID-19 cases have occurred in Chicago. As of April 10, 2020, about 18,000 COVID-19 cases were confirmed out of about 88,000 tests. In Chicago, there are 66 hospitals in which beds are available for the patients and general population (Fig. 2a). The hospitals within 15 miles of buffer zone (about 30 min travel time) from the Chicago boundary were included [24], which helps to take into account that people living near the Chicago boundary may seek hospitals near to Chicago. As of April 10, 2020, there were about 7600 confirmed COVID-19 cases (Fig. 2b).

CDC’s SVI can serve as an indicator of vulnerable populations in the USA based on socioeconomic and demographic characteristics at the census-tract level with four themes: socioeconomic status, household composition and disability, minority status and languages, and housing and transportation (Table 1) [36]. SVI scores range from 0 to 1 with a value of 1 indicating the most vulnerable.

**Table 1:** Social vulnerability index theme and variables

SVI theme	Variables
Socioeconomic status	% Below poverty level % Unemployed Per Capita Income % Age 25 or older with no high school diploma
Household composition & disability	% Age 65 or older % Age 17 or younger % Single parent household
Minority status & languages	% Minority % Age 5 or older speak english “less than well”
Housing & transportation	% Multi-unit structure % Mobile homes % Crowding (more people than rooms) % Households without a vehicle % In institutionalized group quarters

### Parallel enhanced two-step floating catchment area (P-E2SFCA) method

**Fig. 3:**
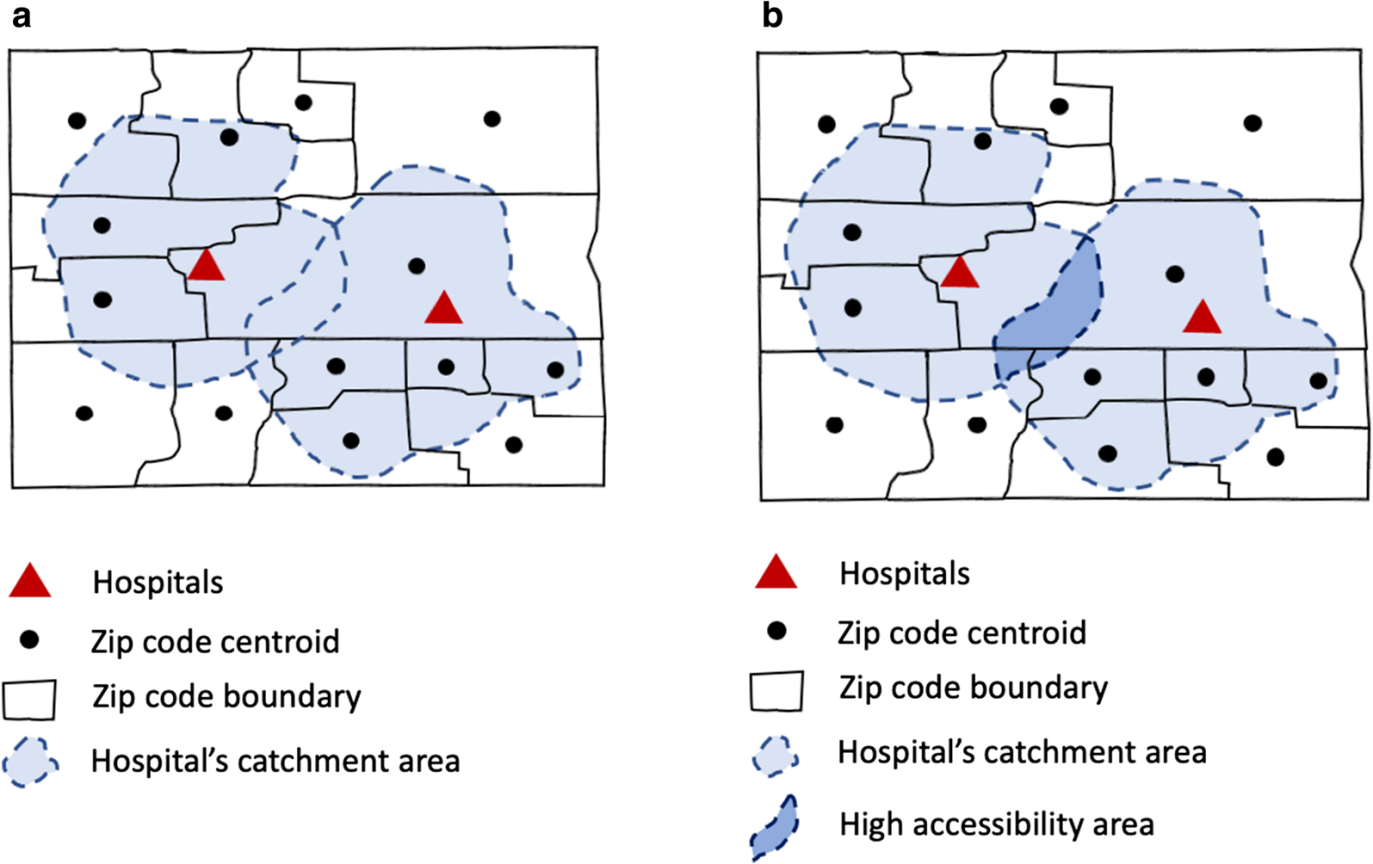
Measure spatial accessibility of a set of hospitals: **a** delineate the catchment area of each hospital; and **b** sum up the accessibility value at residential locations

The conventional two-step floating catchment area (2SFCA) method is based on a service-to-population ratio computed in two steps [24, 43]. First, it is to find all people (*i*) located within a catchment area of each healthcare (*j*), as shown in Fig. 3a. A catchment area is based on a threshold travel time (*d*_*0*_). Then, a service-to-population rate $R_{j}$ is computed within a catchment area.

1 $$R_{j} = \frac{{S_{j} }}{{\mathop \sum \nolimits_{{k \in \left\{ {d_{ij} \le d_{0} } \right\}}} P_{k} }}$$

where for the number of available resources*(s)* at each healthcare (*j*), find all population locations (*k*) that fall within a threshold travel distance (*d*_*0*_).

Then, the accessibility $A_{i}$ at a residential location *i* is computed by summing up the service-to-population ratios, as shown in Fig. 3b.

2 $$A_{i} = \mathop \sum \limits_{{j \in \left\{ {d_{ij} \le d_{0} } \right\}}} R_{j}$$

where *i* denotes a residential location, and $R_{j}$ is the proportion of services per person at healthcare location *j* whose centroids are located within the catchment area.

As a relative measurement, the accessibility measurement $A_{i}$ indicates which geographic areas are relatively more accessible than other areas. For example, overlapped areas in Fig. 3b may have a relatively higher accessibility than other areas. The areas that do not fall within catchment areas of any hospitals have no access to any hospitals within a predetermined travel time (e.g., 30 min).

To fully take into account that people may be more likely to visit closer hospitals than others, an enhanced two-step floating catchment area (E2SFCA) method was developed [25] to resolve the limitation about no distance decay within a catchment area (i.e., residents in the same catchment area are assumed to have equal spatial accessibility). The E2SFCA method accounts for the distance decay, by allowing for multiple travel time zones, such as 0–10, 10–20, and 20–30 min. Three values of weights (1, 0.68, and 0.22) were applied to each travel time zone (0–10, 10–20, and 20–30 min), respectively [25]. Spatial accessibility is therefore computed as a summation of the measure of accessibility at each travel interval.

3 $$A_{i} = \mathop \sum \limits_{{j \in \left( {d_{ij} \in D_{1} } \right)}} R_{j} W_{1} + \mathop \sum \limits_{{j \in \left( {d_{ij} \in D_{2} } \right)}} R_{j} W_{2} + \mathop \sum \limits_{{j \in \left( {d_{ij} \in D_{3} } \right)}} R_{j} W_{3}$$

where $A_{i}$ denotes the accessibility of people at location *i* to hospitals, and the proportions of service-to-population $R_{j}$ at healthcare locations *j* whose centroids are located within the catchment area. $W_{r}$ denotes the distance weight for *rth* travel intervals.

**Fig. 4:**
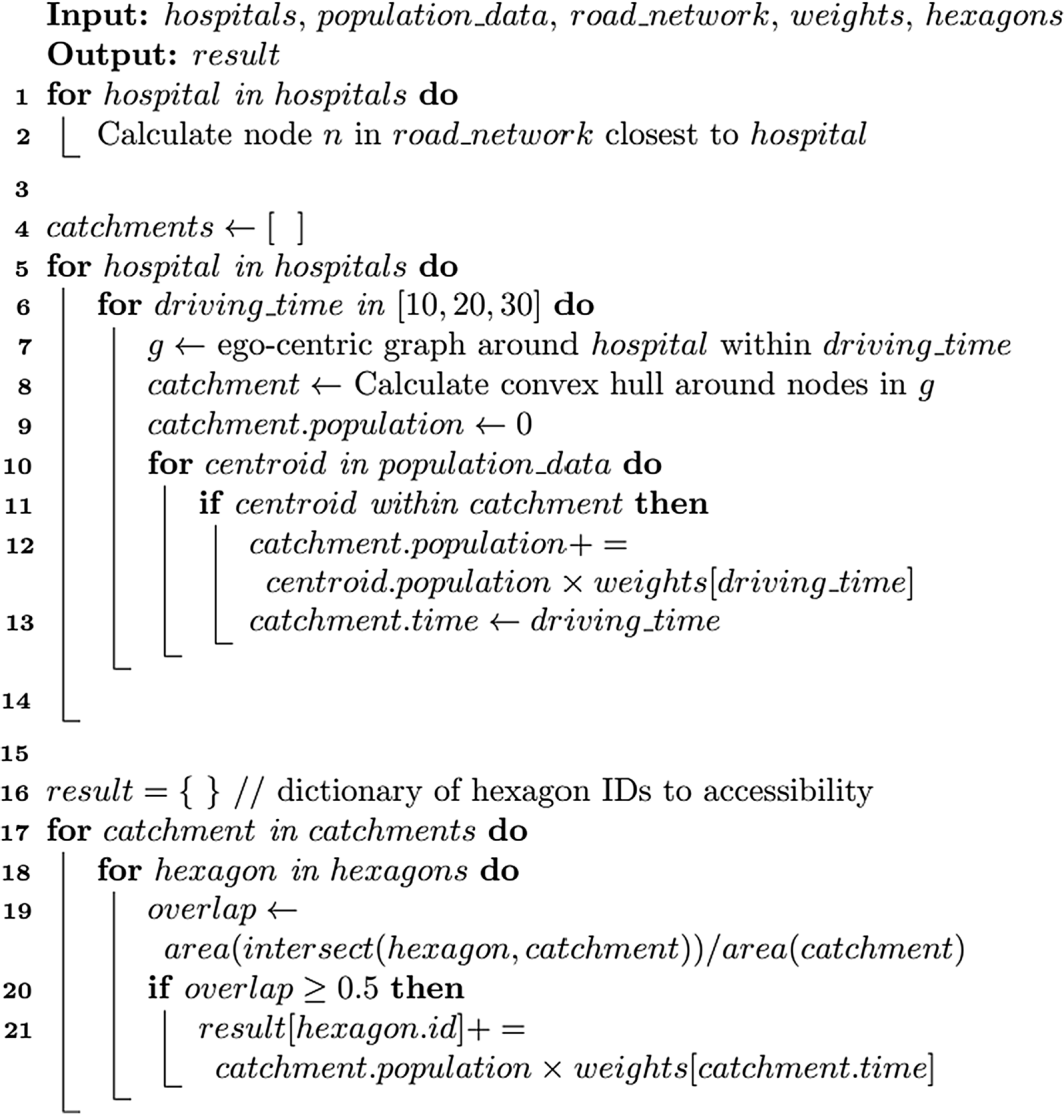
E2SFCA algorithm

**Fig. 5:**
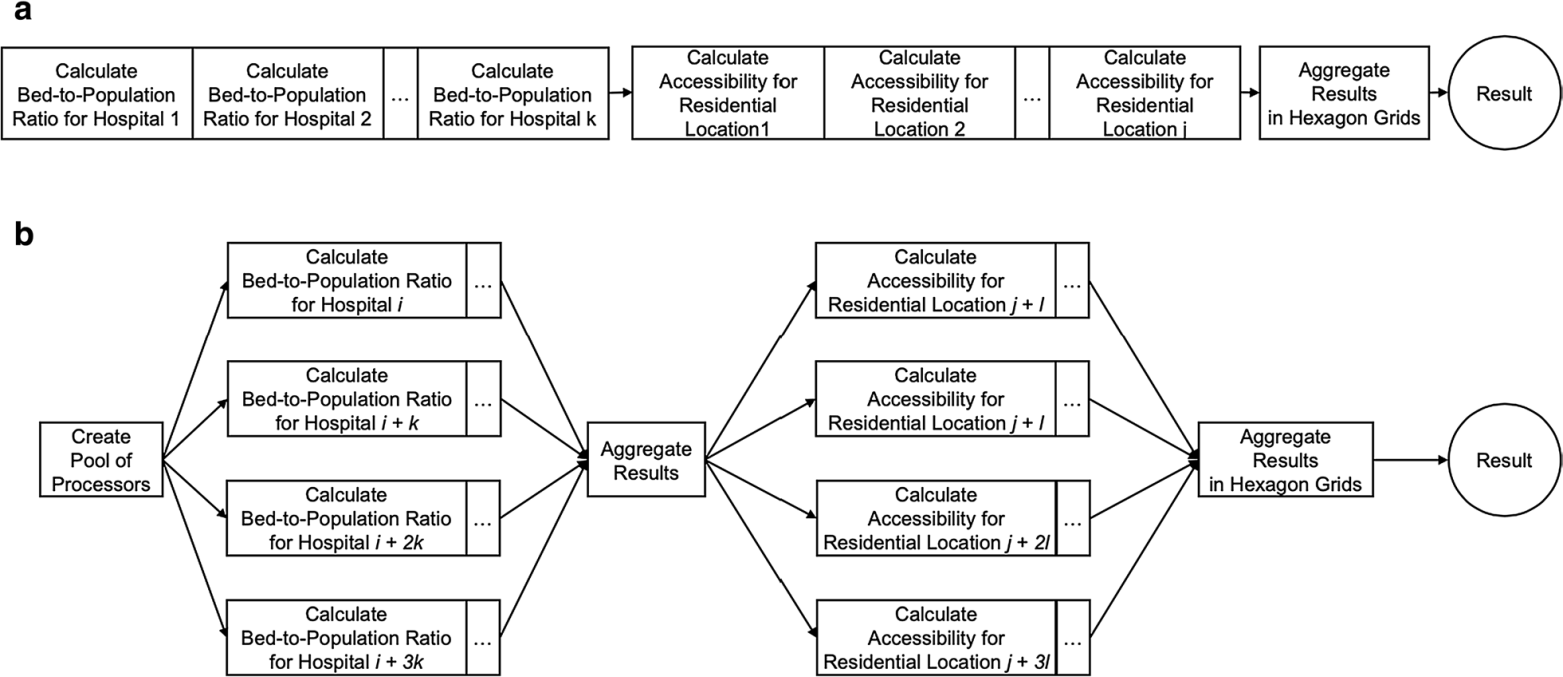
Computational workflows: **a** E2SFCA; and **b** P-E2SFCA

**Fig. 6:**
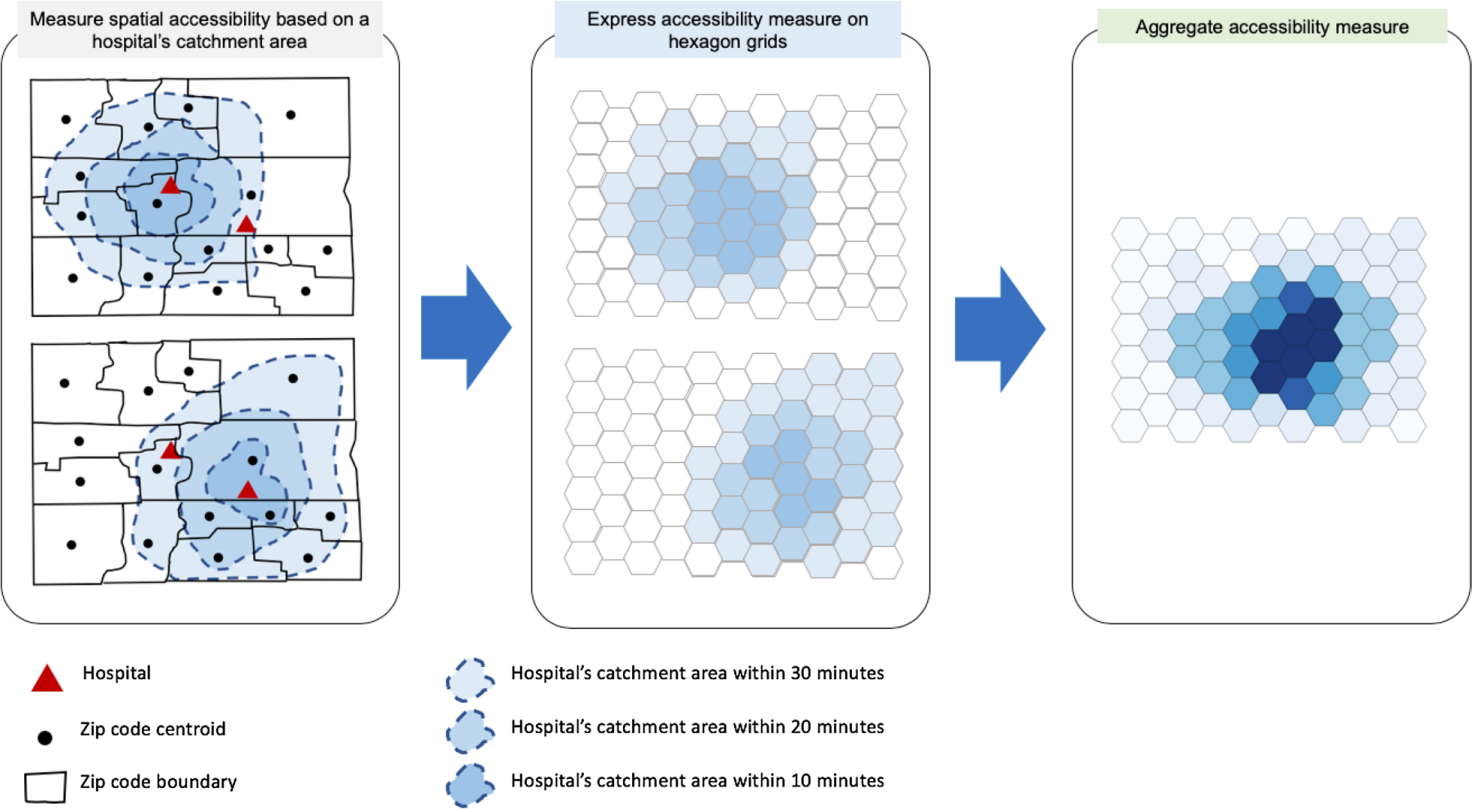
Expression of accessibility measures on hexagon grids. Note: Darker colors represent areas with higher accessibility. The values of hexagons are determined by *A*_*i*_ from Eq. (2)

Figures 4 and 5a show the algorithm and workflow of the E2SFCA method using ICU beds and ventilators as a case of healthcare resources, respectively. The method has two major steps: calculating an ICU bed (or ventilator)-to-population ratio for each supply location (lines 5–14 in Fig. 4) and then summing these ratios for residential locations where supply regions overlap (lines 15–20 in Fig. 4). Since we aim to measure the accessibility for population at risk and COVID-19 patients, we calculated a bed-to-population ratio and a bed-to-COVID-19-patients ratio, separately. The first step delineates a hospital’s 30-min driving zone through a convex hull. It is sub-segmented into 0–10, 10–20, and 20–30 min driving zones. These zones are used to calculate a bed-to-population ratio using a weighted sum of residential locations within each hospital’s catchment area following Eq. (3). Lastly, the accessibility measurements are aggregated into hexagon grids (Fig. 6).

To easily aggregate the accessibility measurements of hospitals, we express the accessibility measurement on regular grids, as shown in Fig. 6. To minimize orientation bias from edge effects, we use hexagon grids, instead of rectangular grids. Each hospital’s catchment area within each driving zone can be expressed on hexagon grids. If there are overlapping catchment areas, the values at each hexagon grid are aggregated. Specifically, we used 500-meter hexagon grids for Chicago and 5-km hexagon grids for Illinois. In Chicago, about 400 meters are the shortest distance between zip code centroids. Therefore, 500-meter hexagon grids are sufficient to represent the accessibility measure. Considering the relatively large area of Illinois, 5-km hexagon grids are suitable for depicting the accessibility measure.

The E2SFCA algorithm is computationally intensive. As described in line 7 of Fig. 4, calculating the catchment area of each hospital involves determining an ego-centric network within a specified driving time using a shortest-path algorithm [44] to compute shortest paths from a source node. The time complexity of our algorithm is *O*(C|E| + C|V|log|V|) where C is the number of catchments (number of hospitals times number of travel times), |V| is the number of vertices, and |E| is the number of edges in the OSMnx network [42]. This complexity is derived based on running the Dijkstra’s shortest-path algorithm for each catchment and travel-time. This complexity poses a significant computational challenge because even after removing nodes with no outdegree and small (less than 10 nodes) strongly connected components, the Chicago street network alone has 28,373 nodes and 75,797 edges all with a wide variety of attributes available from OpenStreetMap, which can be collected by using OSMnx python library. This type of network-based analysis is notoriously difficult for GIS and spatial analysis as it has high computational complexity and takes a prohibitively large amount of memory for scaling up to large geographic areas with sizable networks.

We resolve this significant computational intensity challenge by developing a parallel computing approach called P-E2SFCA to achieve rapid measurement of spatial accessibility for serving the purpose of timely decision support. We parallelized the steps of calculating an egocentric graph of the road network within specified travel times, determining the convex hull of the nodes, and calculating the difference between convex hulls to derive 0–10, 10–20, and 20–30 min driving zones (lines 5–14 in Fig. 4). After these steps, we also parallelized the portion of the steps that calculate intersections between the driving zones to aggregate statistics across different hospitals into a holistic measure of spatial accessibility in hexagon grids. An illustrative example of P-E2SFCA with four processors is provided in Fig. 5b. P-E2SFCA uses Python multiprocessing library [45] to support many parallel threads for scaling to large spatial domains.

As a result, accessibility measures are derived. However, the absolute values of the accessibility measures may not be important to revealing which areas have lower accessibility than other areas. Instead of using the absolute value, we converted the absolute values of accessibility measure to the normalized measure as follows:

4 $$NormalACC_{i} = \frac{{Acc_{i} - min\left( {Acc} \right) }}{{max\left( {Acc} \right) - min\left( {Acc} \right)}}$$

where $Acc_{i}$ refers to the accessibility measure at residential location *i*.

To see which areas have relatively more resources given the COVID-19 confirmed cases in Illinois, we used an existing method to measure the percentage difference in model estimation [21, 46]. The spatial accessibility for COVID-19 patients was compared to the accessibility for population at risk by calculating the percentage difference, as:

5 $${\text{Diff}}\_{\text{ACC = }}\frac{{Normal\,ACC_{COVID - 19} - Normal\,ACC_{PopAtRisk} }}{{Normal\,ACC_{PopAtRisk} }}$$

A positive value of the comparison is derived if the normalized accessibility for the COVID-19 patients is larger than that for population at risk, indicating the proportion of oversupply of ICU beds and ventilators. The zero value indicates that the accessibility for the COVID-19 patients and that for population at risk are identical. Otherwise, if the value is negative, then the patients might have struggles to access healthcare resources. Because the values of the comparison are relative measurements, they do not directly quantify the magnitude of issues for particular areas to access healthcare resources.

## Results

We used the P-E2SFCA method to assess the spatial accessibility of ICU beds and ventilators for population at risk and the COVID-19 patients in Chicago and Illinois, USA, as of April 10, 2020. To address the temporal dynamics in the COVID-19 spread and healthcare resources, the rapid measurement of spatial accessibility has been conducted daily and made available on an open platform called WhereCOVID-19 [47]. In addition, the results from the P-E2SFCA method reveal which areas are relatively more or less accessible to hospital beds and ventilators. Our analysis also helps identify the areas in which there are imbalances between the accessibility for the population at risk and that for the COVID-19 patients.

**Fig. 7:**
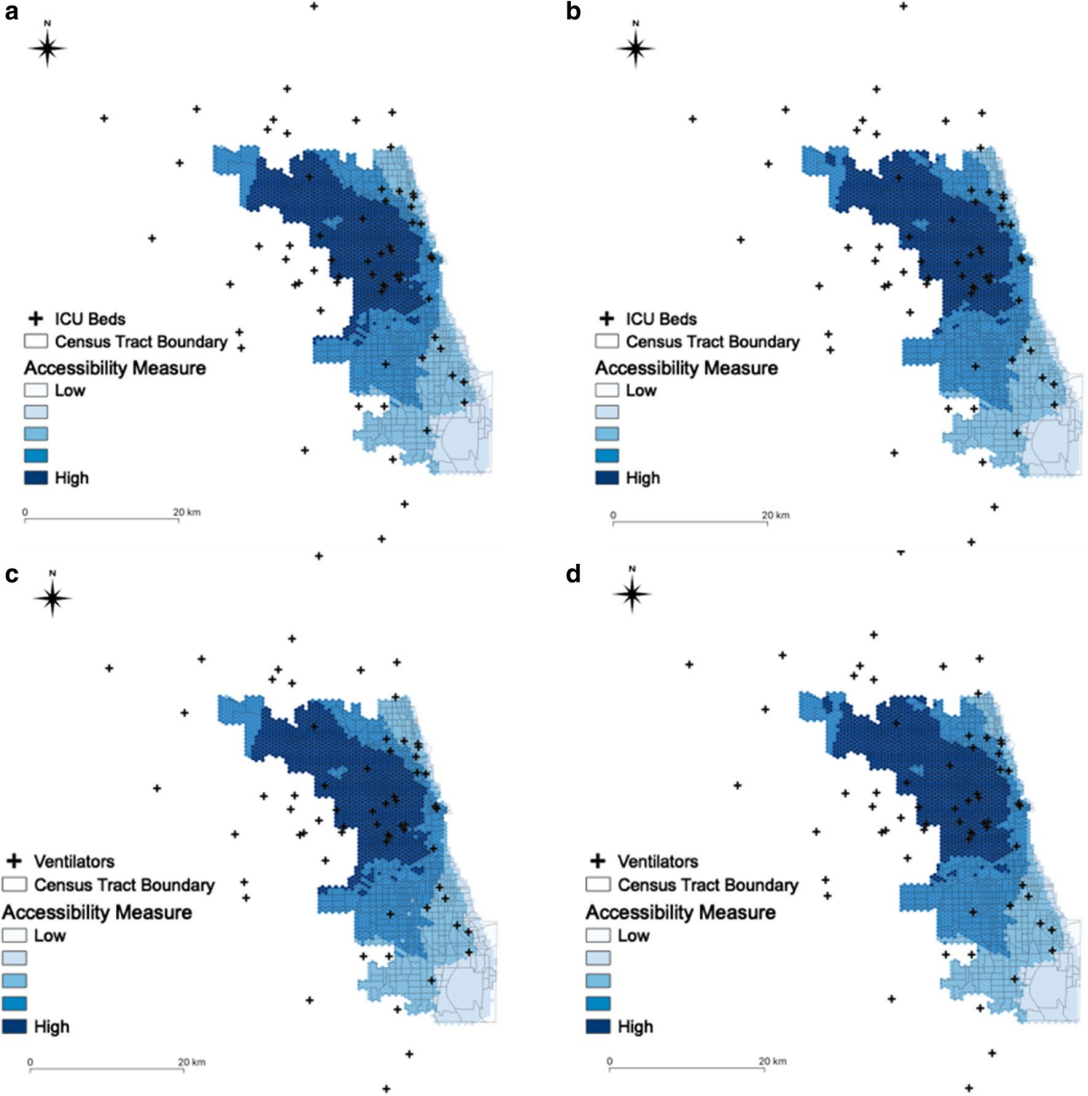
Spatial accessibility measure in Chicago: **a** ICU beds for population at risk; **b** ICU beds for COVID-19 patients; **c** ventilators for population at risk; **d** ventilators for COVID-19 patients

Based on the accessibility measure in Chicago (Fig. 7), we found that the accessibility of hospital beds is spatially varying. The areas with spatial accessibility measures at or above the median are located near the center of Chicago and extending northwestward. The areas with lower accessibility measures are located in southern Chicago. In other words, people living in central and northern Chicago have better accessibility to hospital beds than those living in southern Chicago, as hospitals and hospital beds are mostly located in central Chicago.

**Fig. 8:**
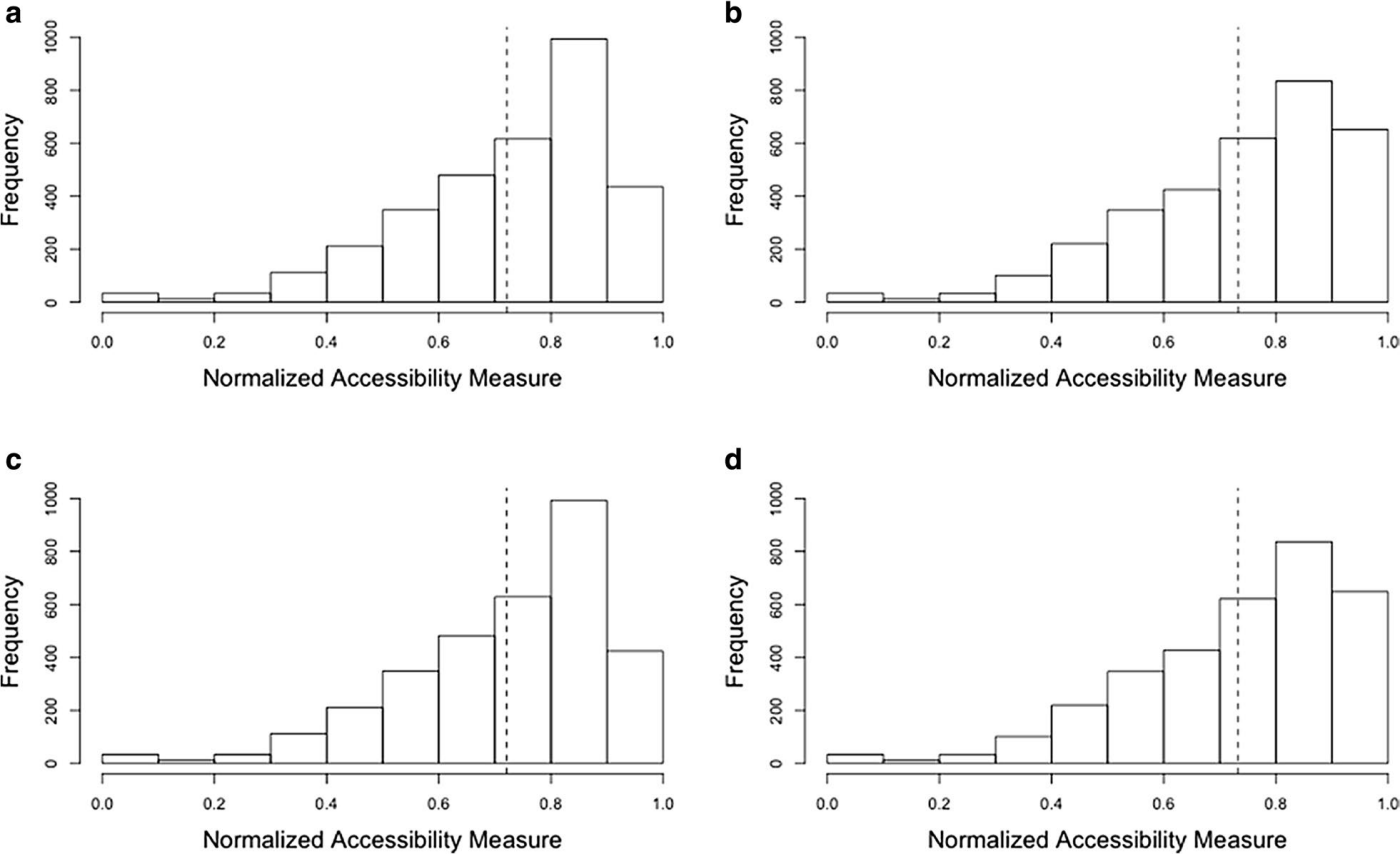
Accessibility measure in Chicago: **a** ICU beds for population at risk; **b** ICU beds for COVID-19 patients; **c** ventilators for population at risk; **d** ventilators for COVID-19 patients. Note: the dotted lines refer to the average of the accessibility measure. Y-axis denotes the number of hexagons, since the accessibility measure is represented through the hexagons

In addition, the difference in access to ICU beds and ventilators for the population at risk and for the COVID-19 patients are statistically significant (*t*_(6551.1)_ = − 2.4713, *p *< 0.05 and *t*_(6551.2)_ = − 2.4896, *p *< 0.05), as shown in Fig. 8. This result implies that access to hospital beds and ventilators was similar for the population at risk and for the patients diagnosed with COVID-19. The median of the normalized accessibility measure of ICU beds and ventilators are equal to 0.7701 for population at risk and 0.7769 for the COVID-19 patients, respectively. In general, ICU beds and ventilators are distributed with high accessibility in Chicago.

**Fig. 9:**
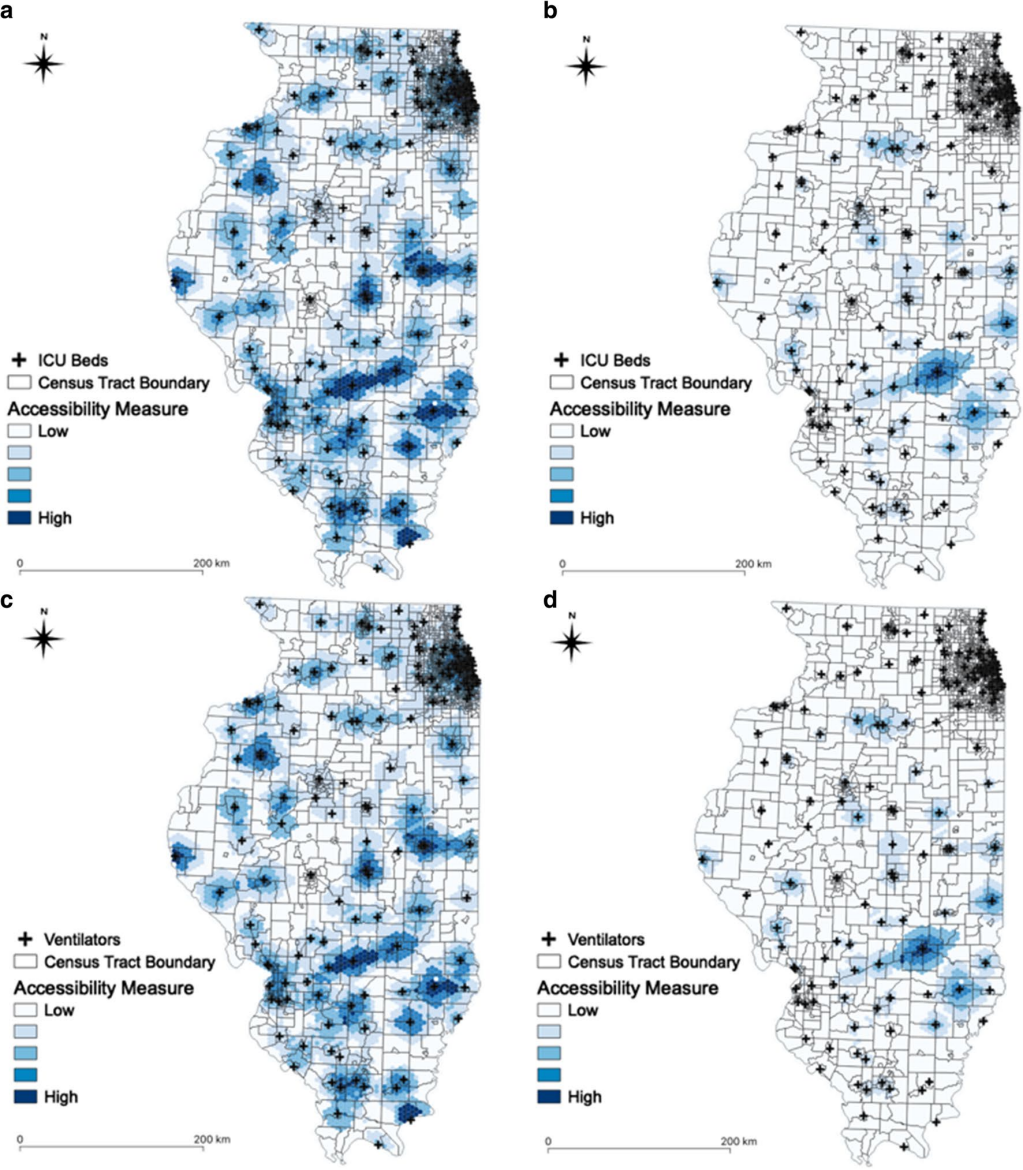
Accessibility measure in Illinois: **a** ICU beds for population at risk; **b** ICU beds for COVID-19 patients; **c** ventilators for population at risk; **d** ventilators for COVID-19 patients

As shown in Fig. 9, the spatial accessibility measure is also spatially varying across Illinois. For population at risk, the areas with higher spatial accessibility are relatively uniformly distributed in Illinois (Fig. 9a, b). On the other hand, only some areas have relatively higher spatial accessibility measures for COVID-19 patients in Illinois (Fig. 9c, d). In central Illinois (e.g., Peoria and Springfield), COVID-19 patients have higher accessibility to ICU beds and ventilators than those living in the other areas of Illinois. We also found that COVID-19 patients have lower accessibility in some areas of Chicago (i.e., northeastern Illinois). While abundant resources are concentrated in Chicago (i.e., northeastern Illinois), the majority of the Illinois COVID-19 cases occurred in Chicago.

**Fig. 10:**
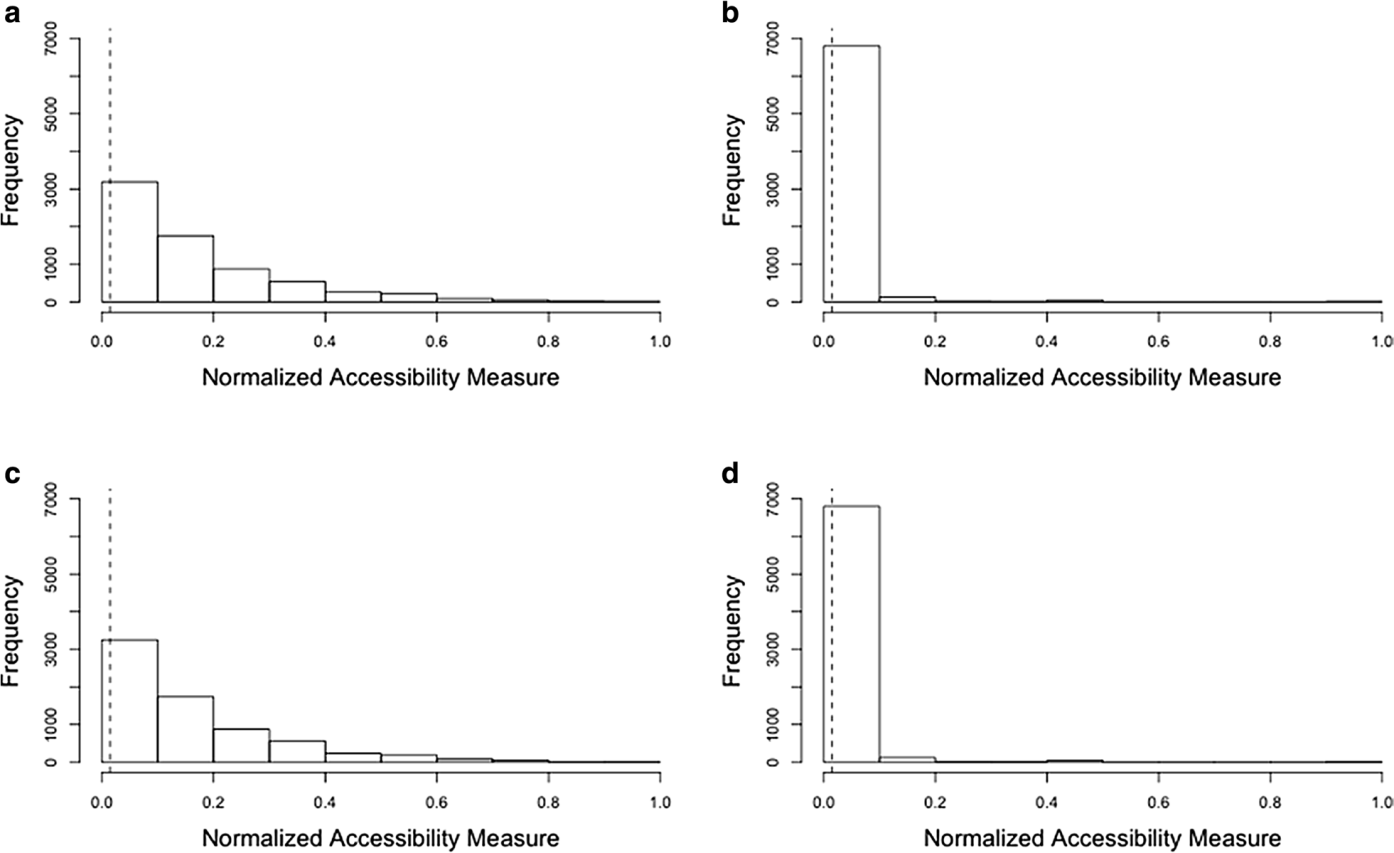
Accessibility measure in Illinois: **a** ICU beds for population at risk; **b** ICU beds for COVID-19 patients; **c** ventilators for population at risk; and **d** ventilators for COVID-19. Note: the dotted lines refer to the average of accessibility measure. Y-axis denotes the number of hexagons, since the accessibility is represented through the hexagons

In Illinois, many residents may not have adequate access to ICU beds and ventilators (Fig. 10). This means that some may be struggling to get access to hospital beds and ventilators at the state level. Population in urbanized areas (e.g., Chicago, Peoria, Springfield) tend to have higher accessibility to ICU beds and ventilators. The median of the normalized accessibility measure is equal to 0.1145 for the population at risk (aged over 50) and 0.0023 for the COVID-19 patients, respectively. The difference in access to ICU beds and ventilators for population at risk and for the COVID-19 patients are statistically significant (*t*_(8589.1)_ = 68.239, *p *< 0.001 and *t*_(8669.8)_ = 68.086, *p *< 0.001).

**Fig. 11:**
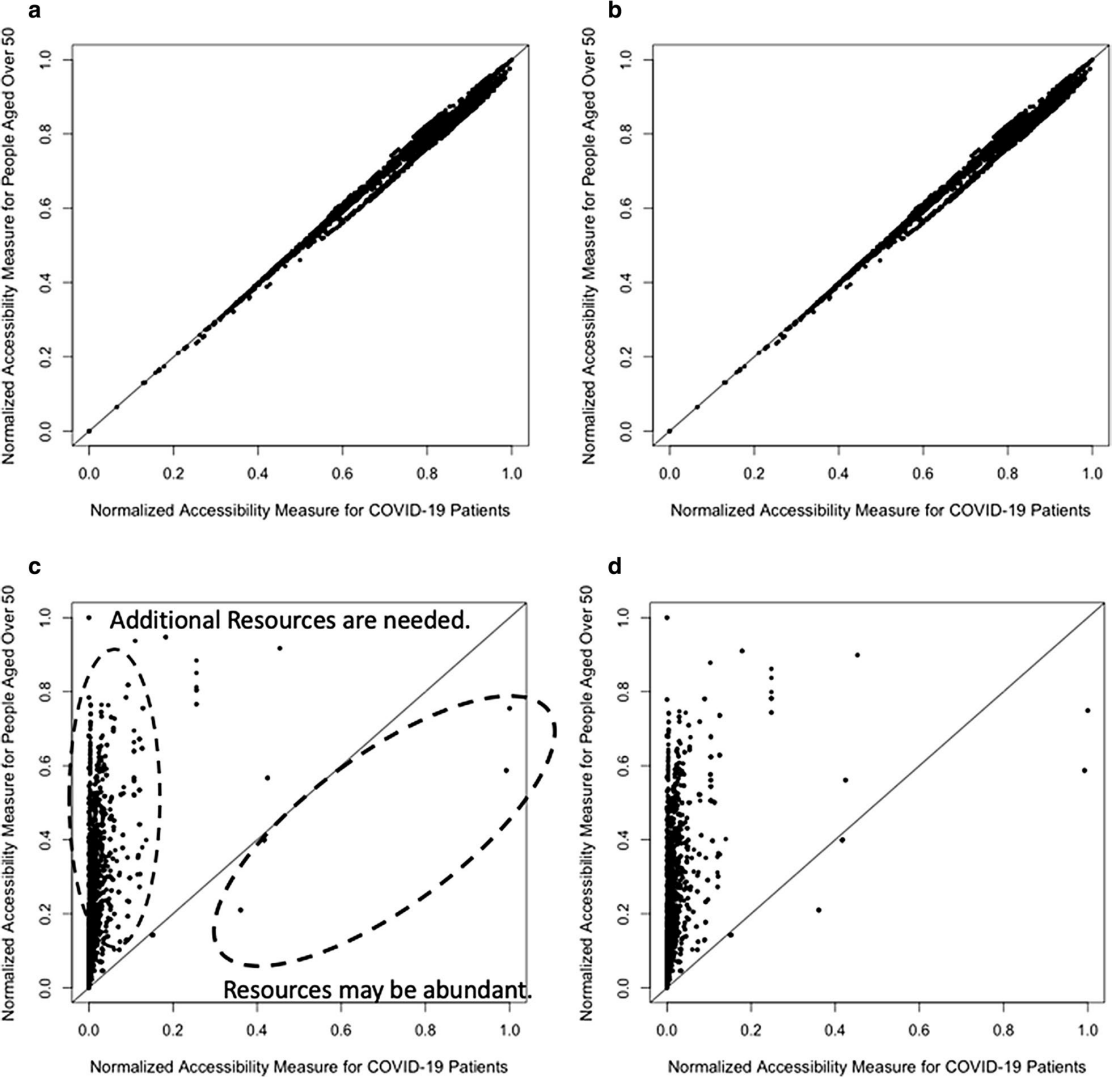
Accessibility measure: **a** ICU beds in Chicago; **b** ventilators in Chicago; **c** ICU beds in Illinois; **d** ventilators in Illinois

Figure 11 presents the scatter plots elucidating whether there are imbalances between the spatial accessibility of ICU beds for population at risk (a) and for COVID-19 patients (b) in Chicago (a), and that of ventilators for population at risk (c) and for COVID-19 patients (d) in Illinois. Each dot represents the accessibility measure at each hexagon. Spearman’s correlation coefficients are 0.9937 (*p* < 0.001) for ICU beds and 0.9937 (*p* < 0.001) for ventilators in Chicago, and 0.7158 (*p* < 0.001) for ICU beds and 0.7107 (*p* < 0.001) for ventilators in Illinois, respectively. If the correlation coefficient is close to one, then there are less imbalances in the spatial accessibility between population at risk and COVID-19 patients. Although the accessibility measures are not significantly different in Chicago, there are some differences in Illinois. This means that some areas may need additional ICU beds and ventilators while such resources may be abundant in other areas. In Fig. 11c, d, the areas having higher accessibility for population at risk, but lower accessibility for COVID-19 patients could be understood as the areas in which additional ICU beds and ventilators may be needed. Furthermore, the number of COVID-19 cases is relatively higher, compared to existing accessible resources in these areas. On the other hand, the areas having higher accessibility for COVID-19 patients, but lower accessibility for population at risk, could be defined as the areas in which ICU beds and ventilators may be abundant.

**Fig. 12:**
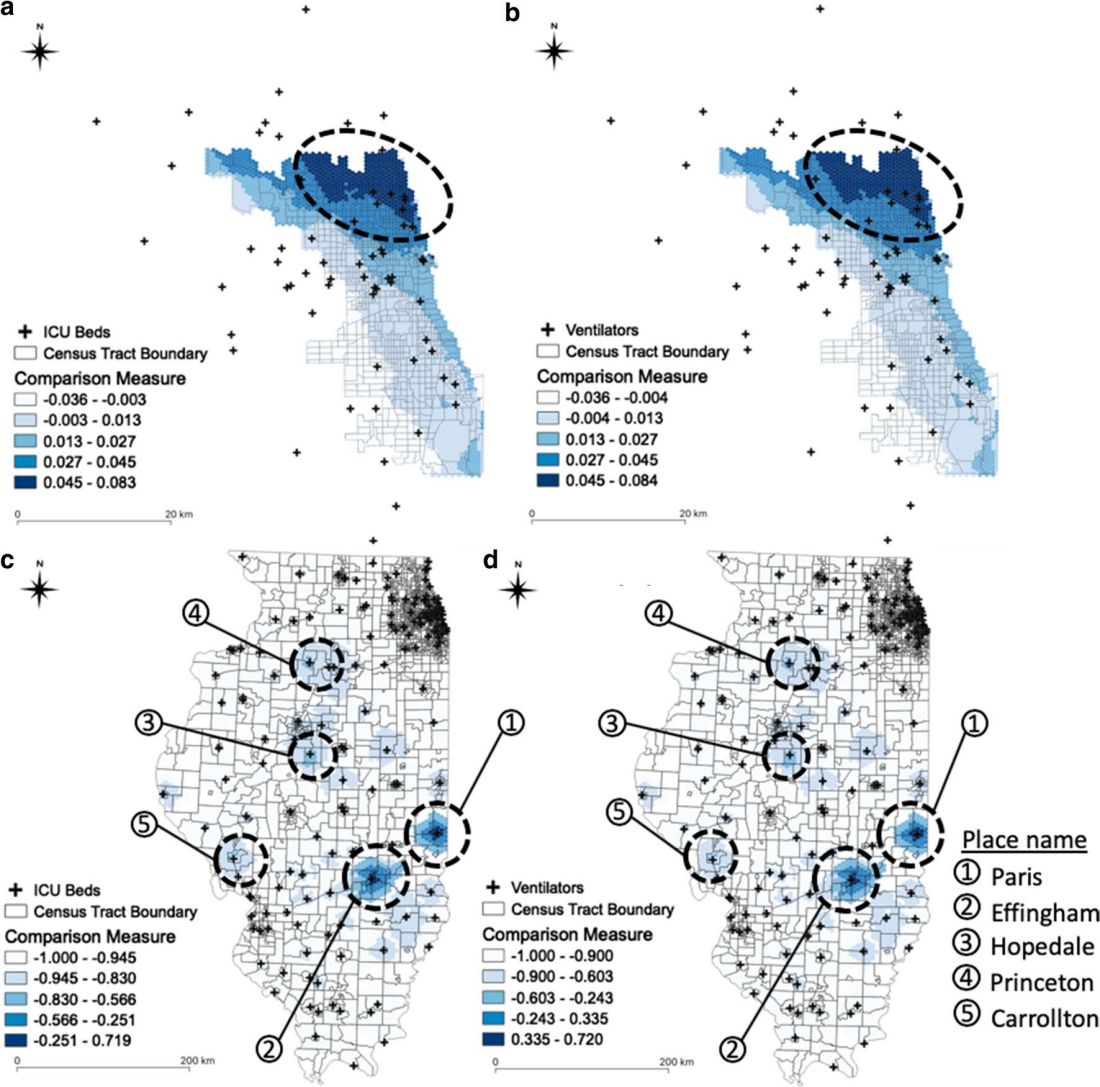
Comparison of spatial accessibility measure: **a** ICU beds in Chicago; **b** ventilators in Chicago; **c** ICU beds in Illinois; **d** ventilators in Illinois

To identify which areas need additional ICU beds and ventilators to resolve the inequality in access to such resources for COVID-19 patients in Illinois, we compared the accessibility measure for COVID-19 patients to that for population at risk, based on Eq. (5). Figure 12 illustrates the measure difference in Chicago for ICU beds (a) and for ventilators (b), and in Illinois for ICU beds (c) and for ventilators (d). The circles identify the areas where ICU beds and ventilators may be abundant. Compared to the accessibility for population at risk, COVID-19 patients have higher accessibility in northeastern Chicago. In other words, it is relatively more difficult for COVID-19 patients living in southwestern Chicago to access ICU beds and ventilators. This also implies that hospital resources are more densely located in northern Chicago. Therefore, it is important to consider increasing the supply of ICU beds and ventilators on the southside via field hospitals and/or reopening recently closed facilities.

At the state level, ICU beds and ventilators are not equitably distributed, especially considering the number of COVID-19 confirmed cases. In Fig. 12 (c) and (d), the circles represent the areas in which ICU beds and ventilators may be abundant, respectively. Paris is the most accessible area for both ICU beds and ventilators, compared to the number of COVID-19 patients, and followed by Effingham, Hopedale, Princeton, and Carrollton. We found that additional ICU beds and ventilators are needed in the Chicago area, despite the substantial number of such resources that are already available. Given that COVID-19 infections mostly occurred in Chicago, additional ICU beds and ventilators may need to be allocated to Chicago, especially the southwestern Chicago area.

### Socioeconomic and demographic characteristics in high and low accessibility areas

To understand the socioeconomic and demographic characteristics in the areas with high and low accessibility of ICU beds and ventilators, we examined the patterns of the socioeconomic and demographic characteristics in Chicago based on spatial accessibility measures as Chicago is the one of the hardest-hit cities in the US. We calculated z-scores for each SVI theme by comparing them to the average scores in Illinois. As a relative measurement, z scores for each SVI theme represent to what extent the people living in high (i.e., upper 25% in accessibility measure) and low accessibility areas (i.e., lower 25% in accessibility measure) are relatively more vulnerable compared to the general population in Illinois. One represents that people are the most vulnerable, and negative one (− 1) represents that people are the least vulnerable.

**Fig. 13:**
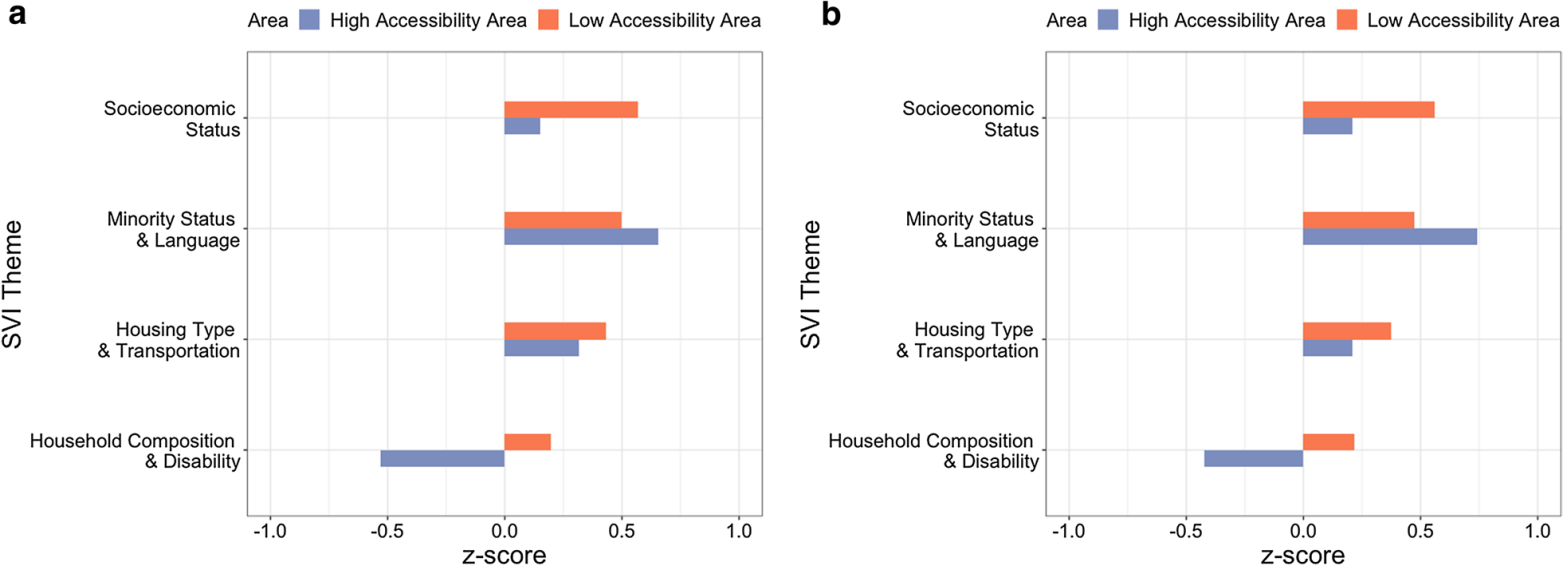
Social vulnerability characteristics in high and low accessibility areas based on the spatial accessibility measure for population at risk (**a**) and that for COVID-19 patients (**b**)

Figure 13a shows the z-scores for people living in high and low accessibility areas in Chicago based on accessibility measures for population at risk, and (b) depicts z-scores for people living in high and low accessibility areas in Chicago based on accessibility measures for COVID-19 patients. The results indicate that people living in areas with low accessibility are more vulnerable to the external stresses, such as the COVID-19 spread, in terms of socioeconomic status, housing type and transportation, and household characteristics and disability. However, it is showed that people living in low accessibility areas are less vulnerable than people living in high accessibility areas in terms of SVI theme on minority status (i.e., racial/ethnic minority) and language (i.e., the proficiency level of English). This can be explained as follows. Given that the SVI theme on minority status and language is a composite score of these two factors, an example of the most vulnerable people would be minority and non-English speakers. For example, the majority of population in a low accessibility area, southern Chicago is African-American and native English speakers. In other words, they are vulnerable in terms of minority status, but not vulnerable in terms of native language. Therefore, this population composition may be responsible for lower vulnerability in low accessibility areas than that in high accessibility areas, in terms of minority status and language theme in SVI.

### Computational performance

We assessed the computational performance of P-E2SFCA using the runtime in seconds of both P-E2SFCA and E2SFCA implementations and ran both sets of code 10 times each to calculate an average runtime in seconds. The computational experiments were conducted using CyberGIS-Jupyter and Virtual ROGER [29, 48]. For computing the accessibility in Chicago, P-E2SFCA executed at 6 times faster (i.e., an average of 942 s across ten runs to an average of 147 s) using 4 computing cores not only by parallelizing computationally intensive parts of E2SFCA, but also by efficiently aggregating results returned from parallel computing resources.

## Concluding discussion

Although extensive studies have focused on forecast [49] and exploration of space–time patterns and trends of COVID-19 cases [50, 51], research questions about the availability and capacity of healthcare resources (e.g., hospital beds, ICU beds, ventilators, healthcare personnel, and testing resources) for treating COVID-19 patients need rigorous investigations. This study has addressed the question of to what extent the population at risk and COVID-19 patients in Illinois, USA have accessibility to healthcare resources. Specifically, we compared the spatial accessibility for the population at risk (aged over 50 years) to that for the COVID-19 patients. The comparison identifies which geographic areas may need additional healthcare resources to accommodate COVID-19 patients.

Specifically, our findings based on the P-E2SFCA method provide an improved understanding of spatial accessibility of ICU beds and ventilators for the population at risk and the COVID-19 patients in Chicago and Illinois, USA, as of April 10, 2020. In addition, our analysis helps identify the areas in which there are imbalances between the accessibility for population at risk and that for COVID-19 patients. The results also reveal which areas have relatively higher spatial accessibility of ICU beds and ventilators. By the comparison of accessibility measures for the general population to that for COVID-19 patients, we found which areas need additional healthcare resources to improve spatial accessibility in Illinois. In general, there are no significant difference in spatial accessibility measures between the population at risk and COVID-19 patients in Chicago. Nonetheless, given that confirmed COVID-19 cases in southern Chicago may exceed the capacity of available healthcare resources, additional resources should be allocated to southern Chicago. At the state level, some areas have much higher accessibility for COVID-19 patients than for population at risk. Compared to the number of confirmed cases, there are more abundant resources in such areas. On the other hand, the Chicago area needs more resources than other areas, considering much more confirmed cases in Chicago.

Given that the number of COVID-19 cases keeps increasing worldwide, the capacities of ICU beds and ventilators need to be properly managed. Other researchers reported that the needs of hospitalization and ICU beds for COVID-19 patients may exceed the current capacity in a number of US cities if the COVID-19 outbreaks were to take place like in Wuhan, China [52]. In this context, it is important to find appropriate ways to effectively and efficiently monitor and manage the capacities and needs of COVID-19 healthcare resources. Our approach helps to reduce the computation time to solution for rapidly measuring the spatial accessibility to COVID-19 healthcare resources in a dynamic and timely manner. Our study has achieved this important and novel capability by being able to conduct spatial accessibility measurement daily with analysis results made available on an open platform called WhereCOVID-19 [47].

Our approach can be applied to other countries in the context of COVID-19 pandemic. For example, the COVID-19 infections have been dominant in the city of Daegu in South Korea and local government in Daegu has committed to a rapid expansion of hospital beds [53]. The COVID-19 has spread to Seoul and another major city (i.e., Daejeon) [54]. In this regard, our approach to looking at the demand and supply together would be helpful for spatially identifying the shortages of COVID-19-related healthcare resources. One remaining issue is that the dataset of hospital locations and the available number of ICU beds and ventilators at each hospital may not be widely available in many countries to be used in assessing spatial accessibility.

Public health agencies and stakeholders such as IDPH need to determine in a timely fashion where COVID-19 healthcare resources are not adequately provisioned to meet the evolving demand. The dynamic spatial accessibility maps generated using our approach and made available on the WhereCOVID-19 platform have served this critical need. The most common use of the spatial accessibility maps is to identify geographic areas where spatial accessibility to COVID-19 healthcare resources is at the lowest level. Public health officials are therefore alerted to make sure these areas get additional support for improving access to specific types of healthcare resources. Our approach integrates cutting-edge cyberGIS and the state of the art of spatial accessibility measurement to derive such important information in an easy to understand and timely fashion, and thus represents a breakthrough in advancing health geographic knowledge for the fight against the COVID-19 crisis.

This study has several limitations. The accessibility measures near state boundaries might be underestimated because we did not include the hospitals in neighboring states (e.g., Indiana, Wisconsin, Iowa, Kentucky, and Missouri), which Illinois residents might visit. Because there are different population distribution patterns, the catchment size parameter needs to be chosen to account for the difference of such patterns [55]. Therefore, assessing the impact of varying catchment size on the spatial accessibility measure [56] would be worth future research. In addition, the COVID-19 cases may not be exhaustively counted because many cases were not confirmed due to having no or light symptoms [57]. Although our results may help to identify the areas where resources are abundant or insufficient, we did not assess how much resources need to be allocated, which should be another future research topic. To more realistically address the dynamic spatial accessibility issues in the COVID-19 context, it would be desirable to consider near real-time changes of healthcare resources such as the numbers of ICU beds and ventilators.

Given mass testing facilities could support a rapid response against COVID-19 spreads [58], a comprehensive measurement of accessibility of ICU beds, ventilators, and testing facilities would directly address the COVID-19 control and preparedness. Unfortunately, there is no publicly available dataset for the testing capacity at each hospital or other COVID-19-related facilities. While our cyberGIS approach to parallel computing of E2SFCA achieves significant computational performance gains, we found that partitioning of road networks is necessary for scaling P-E2SFCA to spatial domains beyond the state level in the US. Future work needs to be done to determine the most optimal means to resolving the computational intensity, by leveraging network partition algorithms such as Voronoi Clustering based on Target-Shift [59, 60], especially for the purpose of applying the analysis to national and international assessment.

In summary, rapidly measuring spatial accessibility of healthcare resources is critical to the fight against the COVID-19 crisis, particularly for better understanding how well the healthcare infrastructure is equipped to save people’s lives. As U.S. federal and state governments (e.g., HHS, IDPH) have been strongly committed to improving spatial accessibility of healthcare services, measuring spatial accessibility and identifying areas with a shortage of important public health resources in the context of COVID-19 is critical for policymakers and public-health officials’ preparedness and response actions. At the same time, strict quarantine, social distancing, and isolation of known cases by individuals and communities are important to slow down the spread of COVID-19 [61, 62], which in turn, help to address the important spatial accessibility issues.

## Data Availability

The datasets and codes generated and/or analysed during the current study are available in the GitHub repository (https://github.com/cybergis/COVID_19Accessibility) and CyberGISX platform (https://cybergisxhub.cigi.illinois.edu/notebook/rapidly-measuring-spatial-accessibility-of-covid-19-healthcare-resources-a-case-study-of-illinois-usa/).
